# The Prognostic Value of Altered eIF3a and Its Association with p27 in Non-Small Cell Lung Cancers

**DOI:** 10.1371/journal.pone.0096008

**Published:** 2014-04-30

**Authors:** Jie Shen, Ji-Ye Yin, Xiang-Ping Li, Zhao-Qian Liu, Ying Wang, Juan Chen, Jian Qu, Xiao-Jing Xu, Howard Lewis McLeod, Yi-Jing He, Kun Xia, Yuan-Wei Jia, Hong-Hao Zhou

**Affiliations:** 1 Institute of Clinical Pharmacology, Xiangya Hospital, Central South University, Changsha, Hunan, P. R. China; 2 Institute of Clinical Pharmacology, Hunan Key Laboratory of Pharmacogenetics, Changsha, Hunan 8, P. R. China; 3 Department of Clinical Pharmacy, Yijishan Hospital of Wannan Medical College, Wuhu, Anhui, P. R. China; 4 Personalized Medicine Institute, Moffitt Cancer Center, Tampa, Florida, United States of America; 5 State Key Laboratory of Medical Genetics, Central South University, Changsha, Hunan, P. R. China; University of Barcelona, Spain

## Abstract

**Background:**

Over-expressed eukaryotic initiation factor 3a (eIF3a) in non-small cell lung cancer (NSCLC) contributed to cisplatin sensitivity. However, the role of eIF3a in oncogenesis was still controversial. This study was designed to investigate the prognostic impact of eIF3a and p27 in radically resected NSCLC patients.

**Methods:**

The expression levels of subcellular eIF3a and p27 were evaluated immunohistochemically in 537 radically resected NSCLC samples, and another cohort of 210 stage II NSCLC patients. Disease specific survival (DSS) and disease free survival (DFS) were analyzed by Kaplan-Meier method and Cox regression model.

**Results:**

The subcellular expression of eIF3a was strongly correlated with status of p27 (Spearman rank coefficient correlation for cytoplasmic eIF3a and p27 = 0.653, for nuclear staining = 0.716). Moreover, survival analysis revealed favorable prognostic impact of nuclear eIF3a, p27, and the combination high nuclear staining on NSCLC (Hazards Ratio = 0.360, 95%CI = 0.109–0.782, *P* = 0.028). In addition, interaction research between biomarkers and chemotherapy status disclosed cisplatin-based regimen trend to prolong DSS of stage II NSCLC patients with high eIF3a-C (*P* = 0.036)and low p27-N (*P* = 0.031).

**Conclusions:**

Our findings suggested altered eIF3a expression closely correlated with p27 status, and the association was of prognostic value for resected NSCLC. Altered expression of eIF3a and p27 predicted prognosis of NSCLC independently.

## Introduction

Non-small cell lung cancer (NSCLC) accounts for 85% of all lung cancers [1]. Although postoperative chemotherapy (CT) was proved to prolong survival significantly [2], 35-50% of NSCLC still relapsed within five years [3]. Hence, reliable prognostic biomarkers would be of great value both for selecting CT candidates and identifying adjuvant CT after surgery.

Eukaryotic initiation factor 3a (eIF3a), the largest subunit of eIF3 complex, had been identified over-expressing in lung cancer [4], and tumors of cervix [5], stomach [6], esophagus [7], nasopharynx [8], colon [9], and oral cavity [10].

However, the role of eIF3a in oncogenesis was still not clear, as it seemed participate in both the protection from and induction of oncogenesis [11]. Some studies had identified eIF3a to promote oncogenesis by negatively regulating translation of p27/Kip1(p27) [12], [13], a member of cyclin-dependent kinase inhibitors (CKIs) to regulate cell proliferation and cell cycle control [14]–[15]. In view of this, eIF3a was likely to serve as an unfavorable prognostic biomarker for tumors.

On the other hand, our previous studies also observed eIF3a enhanced response to cisplatin-based CT in NSCLC through reduction of DNA repair [16]. Moreover, our preliminary experiment on expression of eIF3a and its several correlated factors disclosed a positive correlation between p27 with eIF3a. Herein, high eIF3a might also be indicative for a sensitive phenotype and predict clinical benefits of NSCLC.

Therefore, we hypothesized the status of eIF3a and p27 was associated, and that might be of predictive value for resected NSCLC patients. This research was designed to assess the correlation among eIF3a and p27 expression and their relationship with clinical characteristics including pathology, patient survival and chemotherapy.

## Materials and Methods

### Patient selection

Inclusion criteria for the study were: (a) histologically confirmed primary lung cancers: squamous cell carcinoma (SCC), adenocarcinoma (AC), and adenosquamous (ASC); (b) with follow-up period of more than 40 months; (c) with adequate specimen of primary tumor; (d)when CT status was analyzed, patients in CT arm should finish at least two cycles platinum-based CT. Exclusion criteria were: (a) received any therapy other than postoperative CT (preoperative neoadjuvant CT, radiotherapy, target therapy); (b) non-radical operation, recurrence or death within 3 months after operation; (c) cancer unrelated death.

All patients were staged at the time of surgery according to the guidelines of the National Comprehensive Cancer Network (NCCN). Tumor and morphological classifications were performed by the WHO recommendations. All available tissue blocks and corresponding H&E stained slides were reviewed for identification.

Survival information was obtained from patients relatives or he/she self by telephone follow-up and drop-in on residence registration. The participants provided verbal informed consent due to the pathogenesis of cancer. The patients' records and biomarker analyses were carried out with ethics committee approval by the medical ethical board of Clinical Pharmacology Institute of Central South University. The written consent from patients was waived by the approving institutional review board in this study.

### Immunohistochemistry (IHC) and scoring

Antibodies of eIF3a and p27 were obtained from Cell Signaling Technology (CST, Beverly, Massachusetts) and validated by IHC and WB. The dilution of antibodies was as follows: eIF3a (1∶400), p27 (1: 200). Staining was performed with UltraSensitiveTM S-P ABC kit (Maixin Bio, Fuzhou, China). Negative controls were prepared by replacing the primary antibody with PBS; meanwhile, positive controls were prepared by staining the known-positive specimens of NSCLC in the pathology specimen bank.

All staining specimens were evaluated and scored by two pathologists (H.Y and R. Z) independently without knowledge of patient clinical data. In 10.3% cases (77/747) with discrepancy between 2 observers, the disagreements were reviewed a second time, followed by a conclusive judgment by both pathologists.

An H score was calculated by multiplying the staining intensity (negative, 0; weak positive, 1; positive, 2; strong positive, 3) with the percentage of positive tumor cells. The median H score of each biomarker was used as cutoff criterion. Patients were divided into low group (< median H score) and high group (≥ median H score)[17]. The cytoplasmic and nuclear staining of eIF3a and p27 were scored separately (cytoplasmic eIF3a named as eIF3a-C, eIF3a-N for nuclear eIF3a; p27-C for cytoplasmic p27, and p27-N for nuclear p27).

### Statistical analyses

Disease specific survival (DSS) and disease free survival (DFS) were analyzed in this study. Chi-square tests and multivariate logistic models were used to assess statistical significance of the association of biomarkers with clinicopathologic parameters. Relationship between eIF3a and p27 was studied using Spearman rank correlation on H scores. Survival curves were estimated by Kaplan-Meier method. Log-rank test was used to compare survival time between groups. A Cox proportional hazards model was created to identify independent predictors of survival by using a stepwise selection method (likelihood ratio, backward), with adjustment of all clinical factors. All analyses were performed using SPSS 18.0 software package. Statistical tests were two-sided, and P <0.05 was considered statistically significant for survival analysis.

## Results

After excluding noninformative samples (such as unrepresentative, without valid internal controls), finally we studied informative 537 NSCLC patients undergone curative surgical treatment in the Xiangya hospital (research cohort) between Dec 2007 and Nov 2009. The research cohort consisted of 446 males and 91 females. 168 were identified as stage I, 229 stage II, 140 stage III diseases respectively (table 1). For validation, 218 of 229 stage II patients from research cohort with CT data were brought in predictive analysis (validation cohort), combined with 210 stage II patients from another independent cohort, the Second Xiangya hospital.

**Table 1:** Patients Characteristics And Biomarkers (research cohort n = 537).

		eIF3a-C		eIF3a-N		p27-C		p27-N	
		low	high	low	high	low	high	low	high
Gender	M	199	247	256	190	283	163	253	193
	F	38	53	64	27	51	40	55	36
Age	<55	88	110	108	90	108	90	99	99
	56-64	94	128	128	94	144	78	141	81
	>64	55	62	84	33	82	35	68	49
Smoking status	no	75	86	108	53	106	55	85	76
	Yes	162	214	212	164	228	148	223	153
Stage	I	73	95	104	64	102	66	106	62
	II	104	125	135	94	144	85	129	100
	III	60	80	81	59	88	52	73	67
Histotype	SCC	126	221^b^	208	139	210	137	211	136
	AC	95	75	97	73	113	57	87	83
	ASC	16	4	15	5	11	9	10	10
Nodal status	no	118	134	155	97	146	106	157	95
	yes	119	166	165	120	188	97	151	134
Chemotherapy^a^	no	97	159^b^	139	117	152	104	161	95
	yes	128	135	168	95	172	91	139	124
Differentiation	Well	121	184^b^	174	133	192	115	160	147^b^
	Bad	116	114	146	84	142	88	148	82

a : Chemotherapy data of 18 patients in research cohort were missed.

b : Chi-square test, *P*<0.01 was considered statistically significant (2-tailed).

### Relationship between biomarkers and clinical variables

**Figure 1:**
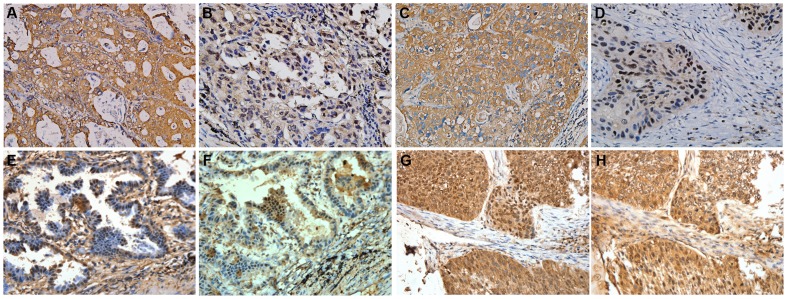
Representative images of eIF3a and p27. A. positive cytoplasmic with negative nuclear staining of eIF3a; B. positive nuclear with negative cytoplasmic staining of eIF3a; C. positive cytoplasmic with negative nuclear staining of p27; D. positive nuclear with negative cytoplasmic staining of p27; Positive nuclear with negative cytoplasmic staining of eIF3a (E) and p27 (F). Positive staining of both cytoplasmic and nuclear eIF3a (G) and p27 (H). (Magnification: 200 for all images).

**Figure 2:**
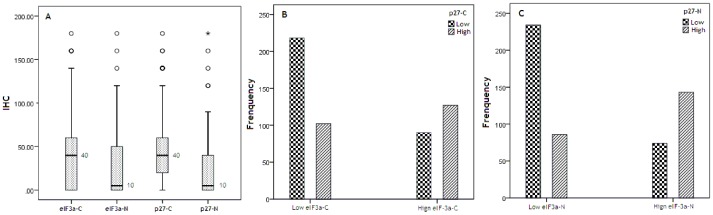
Distribution of subcellular eIF3a and p27. A Distribution of IHC scores in research cohort. (○) Outliers (*) Extremes. B Distribution of p27-C according to eIF3a-C status; chi-squaer test: *P*<0.001. C Distribution of p27-N according to eIF3a-N status;chi-squaer test: *P*<0.001.

In this study, expression of both eIF3a and p27 was primarily a cytoplasmic pattern (fig. 1). High eIF3a was evaluated in cytoplasm of 55.9% (300/537) and in nucleus of 40.4% (217/537) samples (table 1). The Spearman rank correlation coefficient (r_s_) of H scores between cytoplasmic eIF3a and p27 was 0.653, and r_s_ between nuclear eIF3a and p27 reached 0.716. Hence, there was a strong positive correlation between subcellular expressions of eIF3a and p27. Moreover, when patients were divided into low and high group according to H scores of eIF3a and p27 expression respectively, their subcellular expressions were correlated closely: 40.60%(218/537) samples were calculated as low eIF3a-C and p27-C, 23.65% (127/537) as high eIF3a-C and p27-C (fig 2-B); 43.58 (234/537) were low eIF3a-N and p27-N, and 26.63% (143/537) as high eIF3a-N and p27-N (fig 2-C). The correlation was established in validation cohort as well.

Associations of biomarkers with clinical variables were studied (table 1). High eIF3a-C was significantly observed in well differentiated and squamous cell tumors(*P*<0.001). Interestingly, fewer patients with high eIF3a-C tumor received CT treatment(*P*<0.001). P27-N was also observed more frequently in well differentiated tumors (*P*<0.001).

### Prognosis analysis

**Figure 3:**
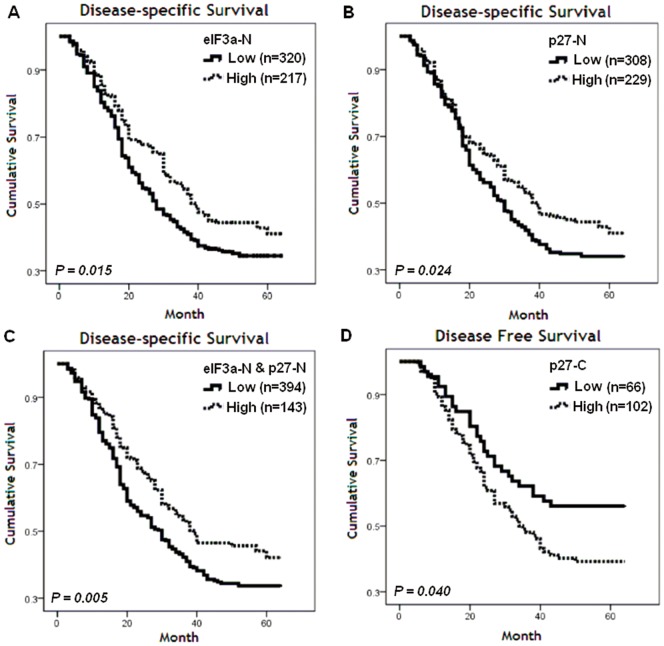
Prognostic analyses according to the expression of eIF3a, p27 status. A. Kaplan-Meier estimates of the probability of DSS according to eIF3a-N (research cohort, n = 537); B. DSS curves according to p27-N(research cohort, n = 537); C. DSS curve according to combination of high eIF3a-N and p27-N(research cohort, n = 537). D. DFS curve of stage I NSCLC according to p27-C in research cohort (n = 168).

The impacts on survival time were investigated according to clinical characteristics and subcellular expression status of eIF3a and p27 (table 2, fig. 3). High eIF3a-N and p27-N was prognostic marker for prolonged survival in univariate analysis (table 2, fig 3-A, B). The prognostic value of eIF3a and p27 was even significant when high eIF3a-N and high p27-N were combined (*P* = 0.005, fig 3-C). Similar prognostic relevance was also clarified in validation cohort (table 3). Moreover, multivariate analysis identified combined eIF3a-N and p27-N as an independent prognostic factor for research cohort as well as for validation cohort. Hazard ratio for death (HR) associated with high eIF3a-N and high p27-N was 0.360 in research cohort (95% CI, 0.109 to 0.782; *P* = 0.028, table 2), and 0.327 in validation cohort (95% CI, 0.143 to 0.794; *P* = 0.011, table 3). At the same time, neither cytoplasmic p27 nor eIF3a was significantly associated with survival of NSCLC (table 2).

**Table 2:** Prognostic Analyses Of Research Cohort (n = 537).

	Univariate analysis			Multivariate analysis		
	Estimate mean DSS(months)	95% CI	*P*(Log rank)	HR	95% CI	*P*
Age			0.328			
<55	37.1	33.9–40.1				
56–64	40.5	37.5–43.6				
>64	38.4	35.7–41.3				
Gender			0.215			
Male	38.7	35.5–40.6				
Female	36.2	33.1–39.5				
Smoking status			0.473			
no	37.8	33.5–40.6				
yes	37.2	33.1–40.1				
Nodal status			0.049^a^			
No	42.0	38.2–45.7				
yes	35.2	31.3–39.0				
Histological type			0.695			
SCC	37.4	34.5–40.0				
AC+ASC	37.6	34.8–40.2				
Differentiation			0.254			
well	36.3	33.2–39.1				
bad	38.5	35.6–40.4				
TNM stage			0.007^a^	1.356	1.028–3.037	0.031^b^
I	41.6	38.7–44.5				
II	37.1	34.0–40.2				
III	33.1	29.8–36.4				
chemotherapy			0.003^a^	2.502	1.130–4.492	0.022^b^
no	40.3	37.5–43.0				
yes	34.6	31.9–37.3				
eIF3a-C			0.353			
low	37.1	34.5–39.7				
high	38.3	35.4–41.1				
eIF3a-N			0.015^a^			
low	35.3	32.8–37.8				
high	40.6	37.6–43.6				
p27-C			0.316			
low	35.7	32.8–39.0				
high	38.9	36.1–41.6				
p27-N			0.024^a^			
low	35.5	33.0–38.0				
high	40.0	37.1–43.1				
eIF3a-N & p27-N			0.005^a^	0.360	0.109–0.782	0.028^b^
low	35.1	32.6–37.6				
high	41.0	38.0–44.0				

a : Univariate analysis (Log-rank method); *P*<0.05 was considered statistically significant.

b : Multivariate analysis (Cox proportional hazard model), *P*<0.05 was considered statistically significant.

Abbreviation: HR: hazard ratio; CI: confidence interval.

**Table 3:** Predictive Analyses Of Validation Cohort (n = 439).

	Univariate analysis			Multivariate analysis		
	Estimate mean DSS(months)	95% CI	*P*(Log rank)	HR	95%CI	*P*
Age			0.416			
<55	39.3	34.2–41.8				
56–64	40.2	36.9–43.8				
>64	37.6	34.7–40.5				
Gender			0.335			
Male	37.5	35.5–40.6				
Female	40.0	37.8–42.5				
Histological type			0.251			
SCC	40.2	37.7–43.1				
AC+ASC	38.3	36.1–40.4				
Differentiation			0.254			
well	39.7	37.2–41.5				
bad	38.5	36.6–40.8				
chemotherapy			0.386			
no	38.1	35.1–41.1				
yes	39.9	36.9–42.9				
Nodal status			0.012^a^	3.371	1.124–7.710	0.018^b^
No	42.2	39.0–45.5				
yes	36.5	33.5–39.1				
eIF3a-C			0.301			
low	38.1	35.3–41.0				
high	40.3	37.1–43.5				
eIF3a-N			0.012^a^	0.565	0.217–0.897	0.021^b^
low	35.5	32.3–38.7				
high	42.1	38.3–45.9				
p27-C			0.816			
low	38.9	36.2–41.7				
high	38.7	35.3–42.1				
p27-N			0.031			
low	35.9	32.7–39.1				
high	41.6	37.6–45.4				
eIF3a-N & p27-N			0.007	0.327	0.143–0.794	0.011^b^
low	36.0	33.2–38.9				
high	44.1	39.5–48.8				

a : Univariate analysis (Log-rank method); *P*<0.05 was considered statistically significant.

b : Multivariate analysis (Cox proportional hazard model), *P*<0.05 was considered statistically significant.

Abbreviation: HR: hazard ratio; CI: confidence interval.

Prognostic values of eIF3a and p27 were also investigated according to combination of subcellular expression. The DSS curve of high eIF3a-N combination trended to surpass low eIF3a-N combination, although the difference did not reach the level of statistical significance in valid cohort. Similar prognostic relevance was observed in subcellular expression of p27.

When stratified by tumor stages, high p27-C was associated with poor DFS in the stage I NSCLC patients of research cohort in univariate analysis(*P* = 0.040, fig 3-D). Meanwhile, subgroup analyses did not reveal stratified significance of eIF3a and p27 by stages, histological type, and nodal status set in univariate and multivariate analysis.

### Predictive analysis

**Figure 4:**
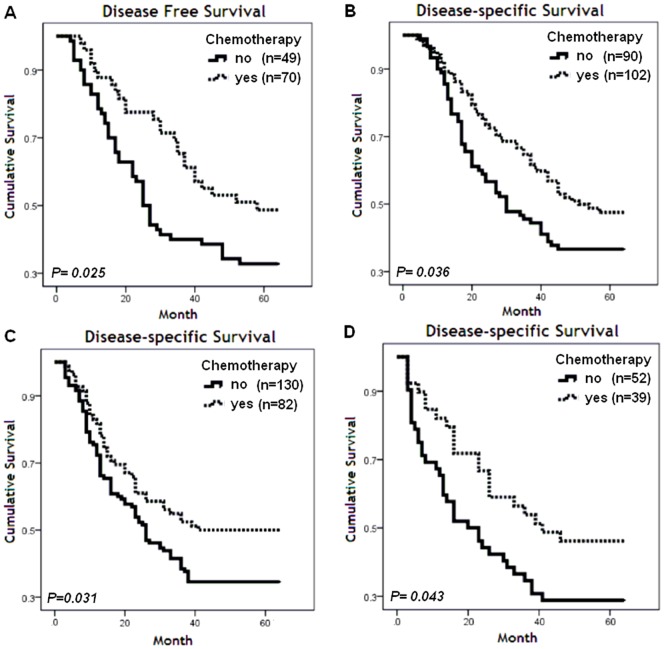
Predictive analyses according to CT treatment and eIF3a, p27 status. **A.** DFS curve of high eIF3a-C subgroup stratified by CT treatment in stage II patients of research cohort (n = 119). B. DSS curve of high eIF3a-C subgroup according to CT treatment in validation cohort (n = 192). C. DSS of low p27-N subgroup according to CT treatment in validation cohort (n = 212). D. DSS of high eIF3a-C and low p27-N subgroup according to CT treatment in validation cohort (n = 91).

In the research cohort, patients accepted chemotherapy (CT) experienced a 6months shorter mDSS than those without CT (table 2), the former usually were associated with higher tumor stages and worse clinical performance status. Howbeit, the survival status and proportion of patients with CT treatment were comparative with those without CT in stage II subgroup (table 3). Additionally, stratified analyses by stages suggested that high eIF3a-C improved DFS associated with CT in such set (*P* = 0.025, fig 4-A), although no biomarker was associated with beneficial CT by DSS in research group. Hence, the predictive analysis were based on stage II patients according to CT status(validation cohort), that including 216 patients received cisplatin-based CT (CT arm) and 212 did not (control arm).

In validation cohort, high eIF3a-C was associated with longer mDSS compared with the low eIF3a-C group, although it did not enter the multivariate analysis (table 3). The interactions between CT and biomarkers were investigated by Cox regression model. CT brought a benefit of 8 months longer mDFS in high eIF3a-C group (table 4, *P* = 0.036, fig 4-B). Furthermore, when analysis was focused on CT treatment, high eIF3a-C also prolonged mDSS compare with low eIF3a-C group (table 4, *P* = 0.025), that was not observed in control arm withal (table 4, *P* = 0.374).

**Table 4:** eIF3a-C predict benefit from CT treatment (validation cohort).

eIF3a-C	CT arm	Control arm	P value
High eIF3a-C	102	90	
Estimate mean DSS (months)	44.7	36.8	
95% CI	40.6–48.8	32.1–41.4	0.036^a^
Low eIF3a-C	114	122	
Estimate mean DSS (months)	36.6	39.6	
95% CI	32.5–40.7	35.7–43.5	0.305
P value	0.025^a^	0.374	

a : Log-rank method *P*<0.05 was considered statistically significant.

CI: confidence interval.

With regard to nuclear staining, neither eIF3a-N nor p27-N retained its significance in CT arm. However, among patients with low p27-N tumors, cisplatin-based chemotherapy brought a 7 months longer DSS than patients from the control group (*P* = 0.031, fig 4-C).

The effect of CT on survival according to combination of eIF3a and p27 expression was also investigated in validation cohort. In combined high eIF3a-C and low p27-N subgroup, mDSS was 8 months longer in the chemotherapy arm than in the control arm (*P* = 0.043, fig 4-D). However univariate and multivariate analysis did not reveal independent predictive significance of such combination. Therefore, eIF3a seemed have a different predictive impact from p27 according to CT status.

## Discussion

Our results indicated an expression-manner dependant prognostic and predictive value of eIF3a: patients with high eIF3a-C or low p27-N would benefit from CT after radical resection of NSCLC; while high eIF3a-N and p27-N was more favorable for overall survival of early stage NSCLC.

Although it was reported that elevated eIF3a, like other eIF3s, would lead to malignant transformation and increase resistance to chemotherapy *in vitro* [18], there were quite some hints that eIF3a might be a favorable predictor of platinum-based CT. Previous studies [5]–[7] indicated high eIF3a predict a better DFS and overall survival. Our previous works showed that eIF3a was associated with well differentiation and SCC, and improved response in lung cancer patients to CT by down-regulating NER proteins [16]. What is more, some recent studies also addressed on the predictive value of eIF3a to cisplatin-based regimen [8], [10], [19]. Hence, an interesting conclusion was eIF3a-C might serve as a new biomarker for selecting CT candidates. Furthermore, the progress on mechanism and regulation of eIF3a might lead to new approaches for antiangiogenesis of tumors [20].

An interesting finding in present study was the subcellular expression of eIF3a closely correlated with p27 status in NSCLC patients, and the prognostic value of such relevance. It had been well established that nuclear and cytoplasmic p27 exert opposite tumorigenic functions [21]. In many cancers, not only p27-N was reduced, but also p27 exhibit different degrees of cytoplasmic localization [22]. It was believed that altered p27 had dual functions in carcinogenesis: reduction of p27-N slackened regulation on cell cycle and increased cell proliferation; what's more, p27-C exhibited cell-cycle-independent oncogenic effects [23]. Interestingly, our study observed chemotherapy brought benefit for patients in low p27, that was also reported by Filipits's work on the IALT program (International Adjuvant Lung Cancer Trial Biologic Program) [17]. Larrea also addressed on post-translational modifications governing p27 localization [24]. The prognostic and predictive relevance of altered eIF3a might also correlate with p27 status in tumors.

Dong *et al* found that eIF3a regulated RRM2 and α-tubulin [25], which also related to cell cycle and proliferation. What is more, expression of eIF3a peaked in S phase [26]. Hence, it could be postulated that altered subcellular localization of eIF3a may play pivotal roles in nuclear exporting as well as in cell cycle regulation. Such presumption merits intensive investigation in future.

Drawbacks may remain insofar due to the retrospective design of predictive study on relationship between biomarkers and CT in present study, such as inadequate randomization. However, stage II population was representative and clinically implicative for CT candidates. As corresponding proportion in either stage I with CT, or stage III without CT treatment, had a relatively low incidence since 2004.

In conclusion, our results suggested that altered eIF3a expression was related to p27 status, and eIF3a could be a potential prognostic factor as well as a marker for efficient treatment of NSCLC.
